# Effectiveness of rotavirus vaccines against rotavirus infection and hospitalization in Latin America: systematic review and meta-analysis

**DOI:** 10.1186/s40249-016-0173-2

**Published:** 2016-08-12

**Authors:** Victor S. Santos, Daniella P. Marques, Paulo R. S. Martins-Filho, Luis E. Cuevas, Ricardo Q. Gurgel

**Affiliations:** 1 Postgraduate Program in Health Sciences, Federal University of Sergipe, Rua Cláudio Batista s/n, Aracaju, Sergipe Zip code: 49060-108 Brazil; 2 Department of Medicine, Federal University of Sergipe, Aracaju, Sergipe Brazil; 3 Investigative Pathology Laboratory, Federal University of Sergipe, Aracaju, Sergipe Brazil; 4 Liverpool School of Tropical Medicine, Liverpool, UK

**Keywords:** Rotavirus vaccines, Rotavirus vaccine effectiveness, Rotavirus genotype, Meta-analysis, Latin America

## Abstract

**Background:**

Rotavirus was the leading cause of childhood diarrhoea-related hospitalisations and death before the introduction of rotavirus vaccines.

**Methods:**

We describe the effectiveness of rotavirus vaccines to prevent rotavirus infections and hospitalizations and the main rotavirus strains circulating before and after vaccine introduction through a systematic review and meta-analysis of studies published between 1990 and 2014. 203 studies were included to estimate the proportion of infections due to rotavirus and 10 to assess the impact of the vaccines. 41 of 46 studies in the post-vaccination period were used for meta-analysis of genotypes, 20 to calculate VE against infection, eight for VE against hospitalisation and seven for VE against severe rotavirus-diarrhoea.

**Results:**

24.3 % (95 % *CI* 22.1–26.5) and 16.1 % (95 % *CI* 13.2–19.3) of cases of diarrhoea were due to rotavirus before and after vaccine introduction, respectively. The most prevalent G types after vaccine introduction were G2 (51.6 %, 95 % *CI* 38–65), G9 (14.5 %, 95 % *CI* 7–23) and G1 (14.2 %, 95 % *CI* 7–23); while the most prevalent P types were P[4] (54.1 %, 95 % *CI* 41–67) and P[8] (33 %, 95 % *CI* 22–46). G2P[4] was the most frequent genotype combination after vaccine introduction. Effectiveness was 53 % (95 % *CI* 46–60) against infection, 73 % (95 % *CI*, 66–78) against hospitalisation and 74 % (95 % *CI*, 68.0–78.0) against severe diarrhoea. Reductions in hospitalisations and mortality due to diarrhoea were observed in countries that adopted universal rotavirus vaccination.

**Conclusions:**

Rotavirus vaccines are effective in preventing rotavirus-diarrhoea in children in Latin America. The vaccines were associated with changes in genotype distribution.

**Electronic supplementary material:**

The online version of this article (doi:10.1186/s40249-016-0173-2) contains supplementary material, which is available to authorized users.

## Multilingual abstract

Please see Additional file 1 for translation of the abstract into the five official working languages of the United Nations.

## Background

Diarrhoea is the second most important cause of childhood death worldwide and rotavirus is the pathogen most frequently associated with severe diarrhoea [1]. More than 90 % of the deaths caused by rotavirus occur in low and middle income countries [2] and in Latin America (LA) alone, rotavirus diarrhoea caused >70 000 annual hospitalisations and 15 000 deaths between 1990 and 2009 [3].

In 2006, two live-attenuated rotavirus vaccines were licensed [4, 5], which was followed in 2009 by the World Health Organization (WHO) recommendation to include them in the national immunization programmes of all countries with high diarrhoea-related child mortality [6]. The vaccines licensed were the pentavalent (G1, G2, G3, G4, P[8]) human–bovine reassortant vaccine (RotaTeq® (RV5); Merck, Whitehouse Station, NJ, USA) and the monovalent (G1P[8]) vaccine derived from an attenuated human strain (Rotarix® (RV1); GlaxoSmithKline Biologicals, Rixensart, Belgium).

The LA region was among the early adopters of the vaccines with 16 countries and one territory introducing at least one of these vaccines in their national immunization programs. National programs have since reported significant reductions in severe rotavirus-diarrhoea episodes, all-cause diarrhoea-related hospitalisations and ambulatory consultations [7–9].

Early reports also described that despite these reductions, a large proportion of rotavirus-diarrhoea episodes were associated with the heterotypic G2P[4] genotype [10, 11]. This is often attributed to a temporal coincidence, as the genotype was circulating in countries with and without rotavirus vaccinations [3]; and to immunological pressure, as the vaccines could have facilitated the selection of genotypes for which they have lower efficacy [12]. Although similar changes have been reported from Belgium [13], Austria [14] and Australia [15]; a systematic review concluded that the genotype selection was unlikely to be due to a selective pressure and that further evidence is needed [16].

Rotavirus vaccines are being introduced in an increasing number of countries and the oldest cohorts of vaccinated children are approaching 10 years. This large scale regional experience has resulted in reports of vaccine effectiveness (VE) to prevent severe diarrhoea and hospitalisations. Recently, a systematic review of reports published between 2006 and 2013 estimated the VE against hospitalisation to range from 63.5 to 72.2 % [17]. This review however did not measure the impact of the vaccines on the burden of rotavirus infection or changes in the frequency of rotavirus strains before and after vaccine introduction.

We conducted a systematic review and meta-analysis to describe the effectiveness of the vaccines to prevent rotavirus infection, hospitalisation and severe rotavirus-diarrhoea in LA and the frequency of rotavirus genotypes reported after vaccine introduction.

## Methods

### Search strategy and selection criteria

We conducted a systematic review using PubMed, the Latin American and Caribbean Health Sciences Literature (LILACS) and SCOPUS databases to identify studies published in Portuguese, Spanish and English between January 1990 and September 2014. Publications were identified using the search terms ≈rotavirus”, “rotavirus infection”, “rotavirus vaccine” and related terms. The full search strategy is described in the Additional file 2. Two independent reviewers (VSS and DPM) screened the title and abstract for relevance. Articles considered to have original material were obtained and assessed in detail.

To assess the proportion of rotavirus in the pre- and post-vaccination periods, we included all observational studies (cohort, case-control, cross-sectional, case series and surveillance) that included children under 5 years of age with symptoms of acute gastroenteritis that had used Enzyme Immune-Assay (EIA) or Enzyme Linked Immune-Assays (ELISA) for the identification of rotavirus. There were no clinical trials in the post-vaccination period. The pre-vaccination period was considered the time prior to the introduction of the vaccine in each country. For example, Brazil introduced the vaccine in March 2006, consequently all data reported from 1990 to before 2006 were considered pre-vaccination.

All studies in the post-vaccination period were included in the description of genotypes if they had used reverse-transcription polymerase chain reaction (RT-PCR). For the description of strain distribution, we included studies reporting the number of samples tested and the G and P combinations.

To evaluate VE against rotavirus infection, we used all studies published in the post-vaccination period and to assess VE against rotavirus-related hospitalizations and severe diarrhoea, we included all case-control studies.

Studies reporting data before and after the introduction of the vaccine were used to assess the impact of the vaccine on the burden of rotavirus disease.

We excluded clinical trials conducted before vaccine licensure, articles without frequencies or percentages of rotavirus-positive children, studies including children with persistent diarrhoea (>2 weeks’ duration), those reporting nosocomial infections, rotavirus B and C infections or limited to outbreaks. There were no clinical trials conducted after the vaccines’ introduction and therefore all studies included were observational.

### Data extraction

Pre-defined tables for data extraction were developed and piloted with 10 papers. The information extracted included author, title, journal, publication year, country, start and end dates, study design, sample size, number of rotavirus-positive and negative samples (overall and by vaccination status), age range, study setting (hospital, hospital and community or community), vaccine type, rotavirus vaccine coverage, proportion of cases due to rotavirus, genotypes identified and frequency. Stool samples with rotavirus and co-infection with other pathogens were considered to be rotavirus-positive. Not all studies reported all variables and percentages were calculated using the number of studies reporting a given variable as the denominator. Countries were classified using the World Bank’s classification for economic development [18] to describe the epidemiological context. To assess VE against rotavirus infection, we extracted the number of vaccinated and unvaccinated children who had rotavirus. To assess VE against rotavirus-related hospitalisation and severe diarrhoea (defined as a Vesikari score >11), we extracted the odds ratio and its confidence interval from case-control studies. The studies’ quality was assessed by two independent reviewers using the Newcastle-Ottawa Scale (NOS) [19].

### Statistical analyses

#### Proportion of rotavirus diarrhoea and genotype distribution

The overall incidence of laboratory-confirmed rotavirus diarrhoea and the proportion of P and G genotypes were calculated using the variance-stabilizing Freeman-Tukey double-arcsine transformation with an inverse-variance random-effects model [20, 21]. We used a Bayesian estimation for genotypes reported as 0 %. To make all proportions different to zero we added 0.5 isolates to the numerator and 1.0 isolates to the denominator. A Pareto chart was prepared to display the strains and cumulative genotype distribution.

The proportion of cases due to rotavirus diarrhoea by country was calculated using the arcsine transformation in a random-effects model. For countries with only one study, the prevalence and 95 % confidence intervals (95 % *CI*) were calculated according to Newcombe’s method [22]. Meta-analysis of single proportions was conducted in RStudio (version 0.98.1083).

#### Vaccine effectiveness

We expressed the protective effect of the vaccines as the relative odds reduction using the formula [100 % x (1-*OR*)]. The odds ratio (*OR*) was defined as the odds of laboratory-confirmed rotavirus infection in vaccinated patients divided by the odds of laboratory-confirmed rotavirus infection in unvaccinated controls.

The overall protective effect of rotavirus vaccination was estimated using the Mantel-Haenszel statistical model. In addition, to assess the VE in preventing hospitalisations due to infectious diarrhoea (any severity) and severe diarrhoea (Vesikari >11), the *OR* and *CI*s were entered in the RevMan software (version 5.3; Cochrane Collaboration) under the generic inverse variance outcome. Forests plots were used to present the pooled *OR* and 95 % *CI*. Two-sided *P*-values <0.05 were considered statistically significant.

Heterogeneity was investigated by the chi-squared test for heterogeneity and quantified using the *I*^*2*^ index [100 % x (Q-df)/Q] [23]. The *I*^*2*^ value ranged from 0 to 100 %, with 25, 50 and 75 % expressing low, moderate and high heterogeneity, respectively. When *I*^*2*^ > 25 %, a random-effects model was applied to estimate the pooled results. Otherwise, the fixed-effects model was used.

Potential sources of heterogeneity were explored by comparing results grouped according to study-level characteristics and by using meta-regression to assess the significance of the differences. The characteristics explored were the vaccine type (RV1 vs. RV5), income (lower middle income vs. upper middle income countries), setting (hospital vs. hospital and community vs. community), latitude, and vaccination coverage. R^2^ index was used to quantify the proportion of variance explained by the covariates [23]. The assumptions of normality, independence, and homogeneity of residuals were verified using diagnostic plots.

Publication bias was assessed using funnel plots of the individual estimates in log units against the standard error and regression tests were performed to analyse the plot asymmetry.

## Results

**Fig. 1:**
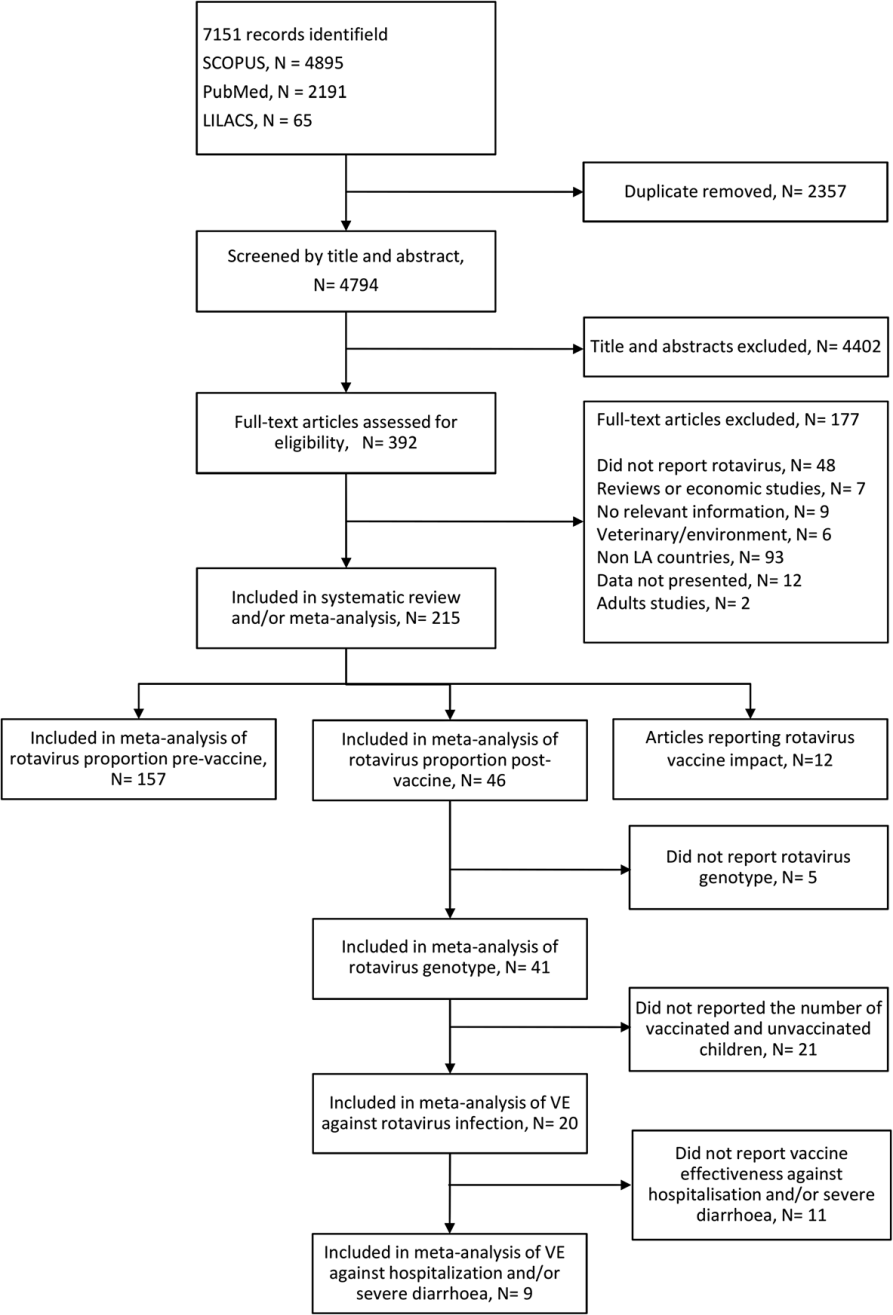
Flow diagram of study selection

The search strategy identified 7 151 records. After screening titles and abstracts, 392 full-text articles were assessed for eligibility and 215 were included. Of these, 203 were used to estimate the proportion of rotavirus, 157 in the pre-vaccine and 46 in the post-vaccine periods. Forty-one of the latter studies were used for genotype meta-analysis. VE was estimated based on 20 studies that reported the number of vaccinated and unvaccinated children. Of these, nine reported data on VE against hospitalisation and/or severe rotavirus-diarrhoea (Fig. 1).

One hundred and thirty-nine (64.6 %) of the 215 studies selected were cross-sectional, 29 (13.4 %) cohorts, 21 (9.7 %) case-control, 14 (6.5 %) surveillance and nine (4.2 %) case series. Two hundred five (95.3 %) studies were hospital-based, eight (3.7 %) hospital and community-based and 15 (6.9 %) community-based (Additional file 2).

### Proportion of rotavirus diarrhoea and genotype distribution

Data extracted from 157 studies in the pre-vaccination period estimated that 24.3 % (95 % *CI* 22.1–26.5) were due to rotavirus. In the post-vaccination period, 46 studies provided data on the proportion of rotavirus cases (Additional file 2: Table S1). Overall, 9 948 (16.1 %, 95 % *CI* 13.2–19.3) of 67,048 children tested for rotavirus infection were rotavirus-positive with the lowest and highest proportion of rotavirus-positive cases being reported from Nicaragua (10.5 %, 95 % *CI* 6.3–15.6) and Mexico (26.7 %, 95 % *CI* 17.1–39.0), respectively. There was high-level heterogeneity across the studies (*I*^*2*^ = 99.1 %, *P* < 0.001). Table 1 describes the proportion of children with rotavirus-positive diarrhoea by country in the pre- and post-vaccination periods.

**Table 1:** Proportion of children with rotavirus diarrhoea pre- and post-vaccination in Latin America

Country	Year of introduction rotavirus vaccine	Vaccine	Pre vaccine		Post vaccine		Difference (%)
			Proportion	*CI *95 %	Proportion	*CI *95 %	
Brazil	2006	RV1	21.1	17.7–24.7	15.8	11.4–20.8	−25.1
El Salvador	2006	RV1	32.9	25.7–40.7	19.7	18.9–20.6	−40.1
Panama	2006	RV1	24.8	3.8–56.4	-	-	-
Venezuela	2006	RV1	21.7	16.9–26.9	-	-	-
Nicaragua	2007	RV5	18.9	14.8–23.6	10.5	6.3–15.6	−44.4
Bolivia	2008	RV1	28.9	16.4–43.3	17.3	15.8–18.9	−40.1
Ecuador	2008	RV1	30.0	20.8–40.1	18.8	15.3–22.9	−37.3
Peru	2008	RV1	24.9	16.7–34.1	-	-	-
Colombia	2009	RV1	29.8	19.1–41.8	18.4	16.1–20.8	−38.3
Honduras	2009	RV1	27.5	14.9–42.3	-	-	-
Mexico	2009	RV1	19.8	12.3–28.5	26.7^a^	17.1–39.0	+34.8
Guatemala	2010	RV1	30.4	13.2–50.9	-	-	-
Guyana	2010	RV1	8.1	5.5–10.1	-	-	-
Paraguay	2010	RV1	25.3	19.4–31.7	-	-	-
Dominican Republic	2012	RV1	61.9	57.1–66.6	-	-	-
Argentina	-	-	26.4	19.9–34.2	-	-	-
Chile	-	-	26.4	19.5–33.9	-	-	-
Costa Rica	-	-	44.7	32.6–57.1	-	-	-
Cuba	-	-	16.6	5.2–32.7	-	-	-
Puerto Rico	-	-	15.6	14.8–16.4	-	-	-
St. Vincent	-	-	25.2	18.1–33.0	-	-	-
Surinam	-	-	33.9	28.3–39.9	-	-	-
Uruguay	-	-	37.2	26.5–48.4	-	-	-

^a^Based on only one study

G and P type information was available for 5 920 and 5 845 isolates from 41 studies (Table 2). Most isolates were reported from Brazil, Nicaragua, and Colombia. G2 was the most prevalent G type (51.6 %, 95 % *CI* 37.8–65.3), followed by G9 (14.5 %, 95 % *CI* 7.4–23.0) and G1 (14.2 %, 95 % *CI* 6.9–23.3). The most common P types were P[4] (54.1 %, 95 % *CI* 41.3–66.5), P[8] (33.2 %, 95 % *CI* 21.9–45.5), and P[6] (3.9 %, 95 % *CI* 1.7–6.7).

**Table 2:** Rotavirus G and P genotype distribution in Latin America, 2006–2014

Genotype	Isolates (*n*)	Proportion (%)	95 % *CI*
G1	1 501	14.2	6.9–23.3
G2	3 170	51.6	37.8–65.3
G3	335	3.6	1.7–6.0
G4	97	0.3	0.0–0.8
G5	33	0.0	0.0–0.2
G8	29	0.0	0.0–0.04
G9	703	14.5	7.4–23.0
G10	1	0.0	0.0–0.2
G12	50	0.8	0.1–1.9
G un-typeable	90	1.2	0.2–2.6
P[4]	3 208	54.1	41.3–66.5
P[6]	327	3.9	1.7–6.7
P[8]	2 265	33.2	21.9–45.5
P[9]	1	0.0	0.0–0.1
P[10]	5	0.0	0.0–0.2
P un-typeable	111	2.2	0.8–4.2

The proportions of genotypes were calculated by using random-effects model

**Fig. 2:**
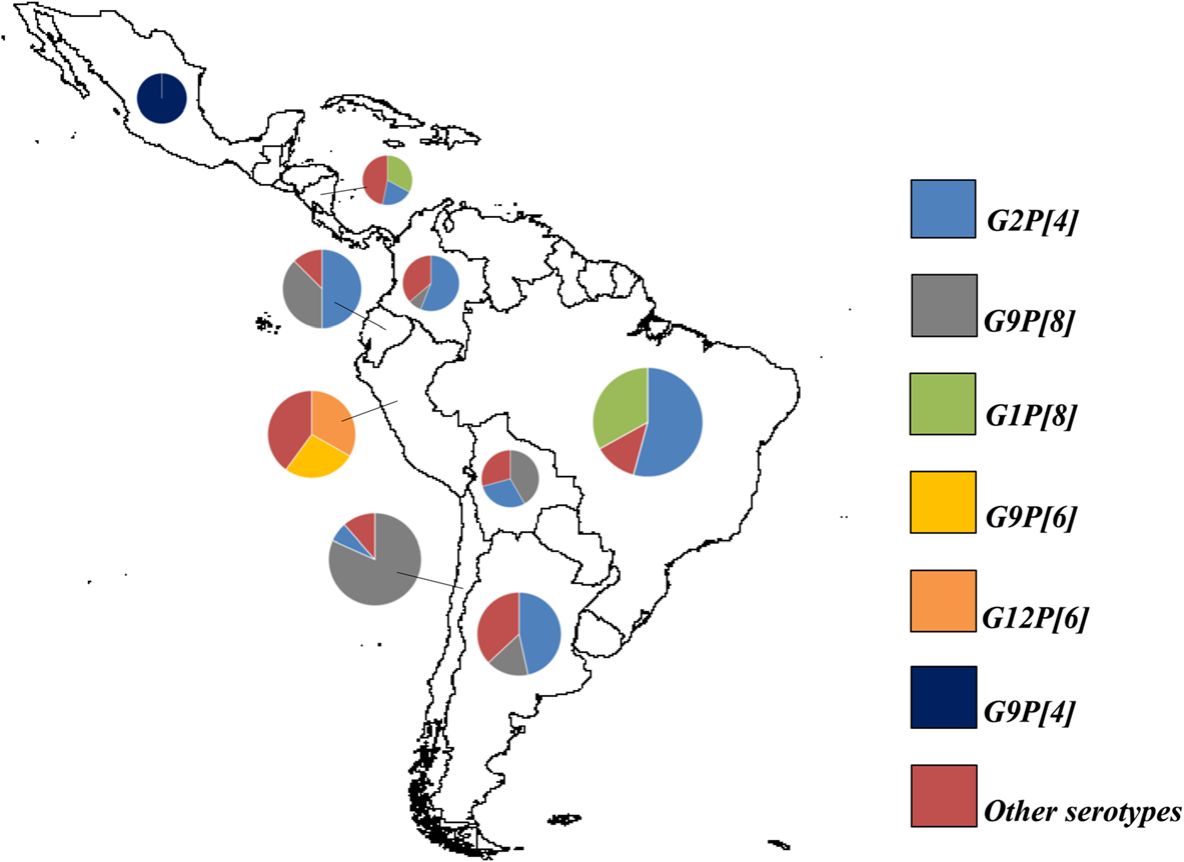
Geographical areas in which rotavirus genotypes are prevalent

G2P[4] was the most prevalent G/P combination in Brazil (54.2 %, 95 % *CI* 32.8–74.9), Argentina (46.6 %, 95 % *CI* 38.9–54.4), Ecuador (50.0 %, 95 % *CI* 33.6–66.4) and Colombia (57.3 %, 95 % *CI* 27.1–84.8) and the second most common combination in Nicaragua (20.3 %, 95 % *CI* 0.2–54.6), Chile (6.8 %, 95 % *CI* 4.0–11.3) and Bolivia (28.9 %, 95 % *CI* 23.7–34.7). The G9P [8] combination was most frequent in Chile (81.7 %, 95 % *CI* 75.6–86.5) and Bolivia (41.8 %, 95 % *CI* 35.9–47.9); and the second most frequent in Argentina (16.4 %, 95 % *CI* 1.3–41.8 %), Ecuador (37.5 %, 95 % *CI* 22.9–54.8), and Colombia (7.8 %, 95 % *CI* 3.0–14.4). G1P[8] (32.9 %, 95 % *CI* 6.2–66.7), G9P[4] (100 %, 95 % *CI* 80.6–100), and G12P[6] (33.3 %, 95 % *CI* 19.2–51.2) combinations were the main genotypes in Nicaragua, Mexico and Peru, respectively (Fig. 2).

### Vaccine effectiveness

**Fig. 3:**
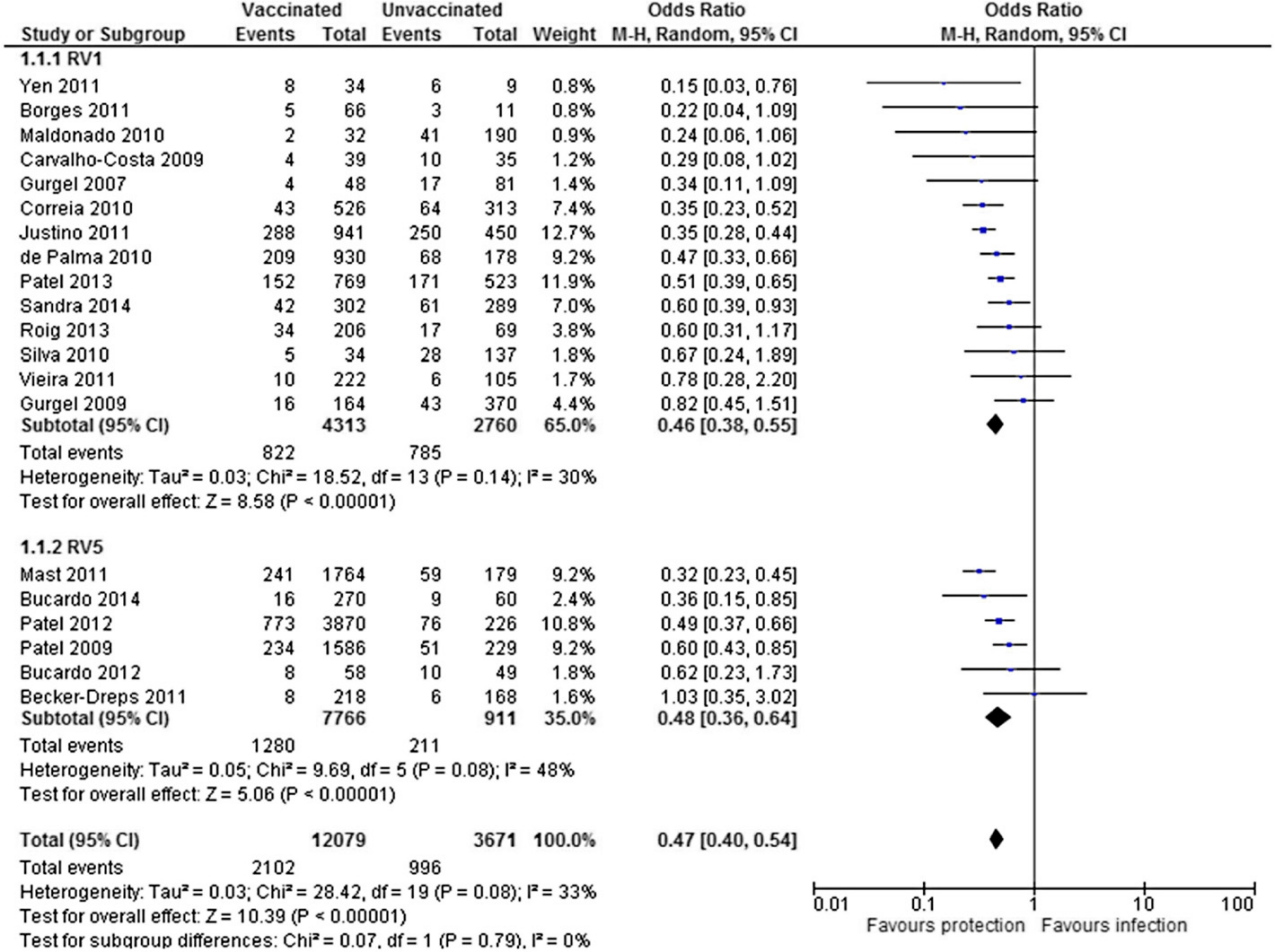
Forest plot of rotavirus vaccine effectiveness against rotavirus infection

Twelve studies from upper-middle income countries (Argentina [24], Brazil [8, 10, 25–31], Mexico [32], and Venezuela [33]) and lower-middle income countries (Bolivia [34], El Salvador [35], and Nicaragua [36–41]), involving 15 750 children were included for the overall analysis of VE. These included 2 102 (17.4 %) rotavirus-positive cases among 12 079 vaccinated and 996 (27.1 %) rotavirus-positive cases among 3 671 unvaccinated children. The overall OR was 0.47 (95 % *CI* 0.40–0.54), resulting in an overall VE against diarrhoea infection of 53 % (95 % *CI* 46.0–60.0). VE was similar for RV1 (54 %, 95 % *CI* 45.0–62.0) and RV5 (52 %, 95 % *CI* 36.0–64.0) (*P* = 0.79) (Fig. 3). There was moderate between-study heterogeneity (*P* = 0.08; *I*^*2*^ = 33 %).

**Fig. 4:**
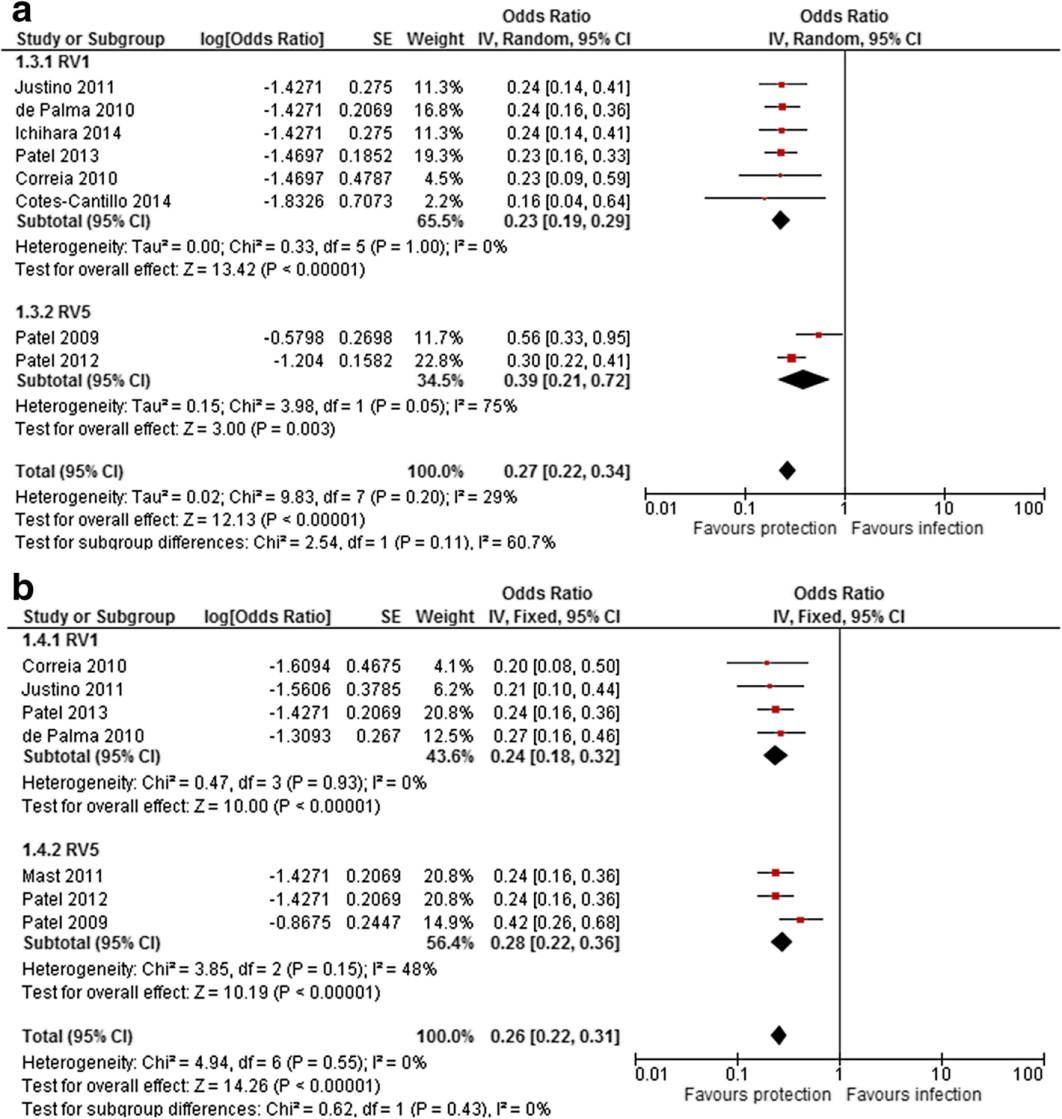
Effectiveness of rotavirus vaccines against rotavirus hospitalisation (**a**) and severe rotavirus-diarrhoea (**b**)

VE to prevent diarrhoea-related hospitalisations (of any severity) and severe rotavirus-diarrhoea was based on eight [26, 34, 35, 40–44] and seven [26, 29, 33, 34, 37, 39, 44] case-control studies, respectively. VE against rotavirus-related hospitalisations was 73 % (95 % *CI*, 66.0-78.0), with moderate heterogeneity among studies (*I*^*2*^ = 29 %, *P* = 0.20). VE against severe rotavirus diarrhoea was 74 % (95 % *CI*, 69.0–78.0) with no evidence of heterogeneity (*I*^*2*^ = 0 %, *P* = 0.55). The level of protection was similar for the two vaccines, as shown in Fig. 4.

To investigate the potential sources of heterogeneity among studies, a meta-regression analysis was performed by using variables as type of vaccine, setting, country income, latitude, and vaccination coverage. Although the difference in protection by latitude was not significant in meta-regression (*P* = 0.258), it was the only factor that partly explained the heterogeneity (adjusted R^2^ = 22.3 %) (Additional file 2: Table S2).

The omission of any of the studies did not modify vaccine effectiveness, suggesting a high stability of the meta-analysis. There was no evidence of publication bias (Additional file 2: Figure S1).

Twelve studies assessed the impact of rotavirus vaccination in countries adopting universal rotavirus vaccination. Of these, five were conducted in Brazil [7, 44–48], four in Mexico [49–52], two in Panama [53, 54] and one in El Salvador [55]. In Brazil, vaccine coverage ranged from 80 % in 2007 to 86 % in 2009. A substantial reduction in deaths (22.0 % to 54.5 % reduction) and hospitalizations (25.0 % to 50.0 % reduction) in children <1 year old was observed, compared to the pre-vaccination period. In Mexico, vaccination coverage remained above 90 % from 2006 to 2011 with significant reductions in deaths and hospitalisations due to gastroenteritis. In Panama, national hospital database studies comparing diarrhoea-related hospitalisations and deaths prior (2000–2005) and after (2008) vaccination reported a 45 % reduction in hospitalisations and 50 % of deaths among infants aged <1 year old. Rotavirus vaccine coverage at that time was above 80 %. El Salvador reported a reduction in hospitalisation rates. Rotavirus vaccine coverage in 2008 and 2009 was 74 and 89 %, respectively (Table 3).

**Table 3:** Impact of rotavirus vaccination on hospitalisation and mortality due to diarrhoea in Latin America

Study	Country	Vaccine	Outcome	Pre-vaccination		Post-vaccination		Difference (%)
Diarrhoea mortality				Year	Results	Year	Results	
do Carmo 2011 [7]	Brazil	RV1	Annual death rates/100 000 children	2002–2005	<1 yr: 48	2007–2009	<1 yr: 35	−22
					1 yr: 11		1 yr: 7	−28
					2–4 yr: 1		2–4 yr: 1	−4
					All: 12		All: 9	−22
Gurgel 2011 [45]	Brazil	RV1	Number of hospitalisation	2002–2005	<1 yr: 986	2006–2009	<1 yr: 449	−54.5
					1–4 yr: 237		1–4 yr: 159	−32.9
					All: 1 223		All: 608	−50.3
Lanzieri 2011 [46]	Brazil	RV1	Annual death rates/100 000 children	2004–2005	<1 yr: 56.9	2008	<1 yr: 34.9	−39
					1–4 yr: 4.5		1–4 yr: 3.0	−33
Richardson 2010 [49]	Mexico	RV1	Annual death rates/100 000 children	2003–2006	<1 yr: 61.5	2008	<1 yr: 25.5	−41
					1–2 yr: 21.1		1–2 yr: 6.1	−29
					2–5 yr: 2.9		2–5 yr: 0.2	−7
					All: 18.1		All: 6.3	−35
Gastañaduy 2013 [50]	Mexico	RV1	Annual death rates/100 000 children	2003–2006	<1 yr: 59.1	2009–2011	<1 yr: 28.4	−52
					1–2 yr: 19.6		1–2 yr: 7.9	−60
					2–5 yr: 2.8		2–5 yr: 2	−26
					All: 17		All: 8.5	−50
Bayard 2012 [53]	Panama	RV1	Annual death rates/100 000 children	2000–2005	<1 yr: 73	2008	<1 yr: 40	−45
					1–4 yr: 20.3		1–4 yr: 9	−54
					All: 31.1		All: 15.5	−50
Diarrhoea hospitalisation								
do Carmo 2011 [7]	Brazil	RV1	Hospitalisation rates/100 000 children	2002–2005	<1 yr: 2 477	2007–2009	<1 yr: 1 840	−25
					1 yr: 2 487		1 yr: 1 886	−21
					2–4 yr: 774		2–4 yr: 722	−7
					All: 1 429		All: 1 165	−17
Gurgel 2011 [45]	Brazil	RV1	Number of hospitalisation	2002–2005	<1 yr: 194 348	2006–2009	<1 yr: 125 151	−35.6
					1–4 yr: 301 479		1–4 yr: 264 376	−12.3
					All: 495 827		All: 389 527	−21.4
Masukawa 2014 [47]	Brazil	RV1	Hospitalisation rates/100 000 children	2000–2005	<1 yr: 255.8	2007–2011	<1 yr: 163.9	−35.9
					1 yr: 241.5		1 yr: 181.3	−24.9
					2 yr: 133.2		2 yr: 118.3	−11.2
					3 yr: 83.4		3 yr: 76.5	−8.3
Fernandes 2014 [48]	Brazil, São Paulo State	RV1	Hospitalisation rates/100 000 children	2000–2005	<1 yr: 1 009.3	2008–2011	<1 yr: 504.5	−50
					1 yr: 743.1		1 yr: 442.5	−40
					2–4 yr: 385.4		2–4 yr: 279.7	−27
					All: 630.8		All: 376.6	−40
Yen 2011 [55]	El Salvador	RV1	Hospitalisation rates/100 000 children	2005–2006	<1 yr: 499	2008	<1 yr: 79	−84
					1–2 yr: 447		1–2 yr: 63	−86
					2–3 yr: 123		2–3 yr: 43	−65
					3–4 yr: 30		3–4 yr: 18	−41
					4–5 yr: 26		4–5 yr: 8	−68
					All: 225		All: 42	−81
Yen 2011 [55]	El Salvador	RV1	Hospitalisation rates/100 000 children	2005–2006	<1 yr: 499	2009	<1 yr: 106	−79
					1–2 yr: 447		1–2 yr: 96	−79
					2–3 yr: 123		2–3 yr: 67	−46
					4–5 yr: 26		4–5 yr: 23	−11
					All: 225		All: 70	−69
Quintanar-Solares 2011 [52]	Mexico	RV1	Number of hospitalisation	2003–2006	<1 yr: 5 133	2009	<1 yr: 6 597	−40
					1–2 yr: 3 944		1–2 yr: 2 441	−52
					2–5 yr: 1 853		2–5 yr: 2 265	−43
Esparza-Aguilar 2014 [51]	Mexico	RV1	Hospitalisation rates/10 000 children	2003–2006	<1 yr: 684	2008–2011	<1 yr: 358	−48
					1–2 yr: 2 301		1–2 yr: 1 195	−48
					2–5 yr: 888		2–5 yr: 733	−18
					All: 945		All: 590	−38
Molto 2011 [54]	Panama	RV1	Number of hospitalisation	2000–2005	<1 yr: 1 359	2008	<1 yr: 941	−31
					1–4 yr: 2 698		1–4 yr: 1 614	−40
					All: 4 057		All: 2 555	−37
Bayard 2012 [53]	Panama	RV1	Number of hospitalisation	2000–2005	<1 yr: 1 062	2008	<1 yr: 762	−28
					1–4 yr: 1 942		1–4 yr: 1 347	−31
					All: 3 004		All: 2 109	−30

## Discussion

Rotavirus-related diarrhoea is still an important public health problem in low- and middle-income countries and the early and widespread use of the vaccines in LA has resulted in a large number of studies and samples analysed, providing an excellent opportunity for their post-licensure evaluation. This meta-analysis estimated that rotavirus VE was 53 % against rotavirus infections, 73 % against rotavirus-related hospitalisations and 74 % against severe diarrhoea episodes. The vaccines (RV1 and RV5) had similar effectiveness. These findings highlight the occurrence of significant reductions of hospitalisations and deaths, as well as decreases in the proportion of diarrhoea episodes due to rotavirus among the countries that adopted universal rotavirus vaccination.

The trials conducted for the registration of the vaccines included a large number of children from middle- and high-income countries from Europe, North America and LA and their main end-points focused on severe diarrhoea episodes and hospitalisation. Their efficacy against severe rotavirus-diarrhoea ranged from 85 to 98 % and rotavirus-associated hospitalisation ranged from 85 to 94 % [4, 5]. In these trials however, vaccine efficacy for all-cause diarrhoea hospitalisation was only 39 % and data on the efficacy of the vaccines to reduce infections was not reported [56]. Our findings reflect real-world outcomes, that are different from those reported under clinical trial conditions and seem to be lower than in Europe, where VE against hospitalisations ranges from 80 to 98 % [57]. There seems to be a variation of the protective effect of the vaccine according to the setting, and trials in low/middle-income African countries have reported a lower efficacy of the RV1 vaccine [58, 59], which could be due to the higher burden of disease in these settings.

This meta-analysis reinforces that the introduction of the vaccines reduces hospitalisations, and reduces the frequency of severe rotavirus episodes and deaths in children <5 years old. The studies summarised provide significant evidence of reductions in hospitalisations and deaths not only in children that received the vaccines, but also in older children. Similar data have been reported from Europe [57] and the United States [9], which may be due to a herd effect of the vaccine enhancing its impact when implemented at large scale under routine conditions. The reduction in hospitalisations and deaths would result in large cost savings [60]. It is estimated that from 2007 to 2025, universal vaccination could avert 141 medical visits for every 1000 children vaccinated in Latin America and save >16 000 lives [61].

Overall, our findings show a decrease in the proportion of diarrhoea infections by rotavirus in the post-vaccine era and provide further evidence that rotavirus vaccinations are associated with a reduction in rotavirus-diarrhoea morbidity. The 16 % proportion of children with rotavirus infection is much lower than the proportion reported before vaccination introduction (range 24–47 %) [3, 62, 63], but varied considerably among countries. This variability may be explained by differences in the burden of disease across study settings, the case definitions used; that some countries were represented by only one study and that some studies were conducted soon after vaccine introduction, which complicates the interpretation of data.

Historically, rotavirus genotypes before vaccines introduction varied over time and the peak frequency of one strain was often followed by a trough and replacement by a different genotype [64]. The strains found most commonly in LA before vaccines introduction were G1P[8], G9P[8] and in a lower proportion G2P[4] and these strains were similar to the most frequent genotypes reported worldwide [3, 64, 65]. After the introduction of the vaccines, a high proportion of studies reported that the highest number of cases were due to the G2P[4], especially in countries that adopted the RV1 vaccine. Similar changes were observed in Oceania [15] and Europe [13, 14, 66]. Latin American countries that did not adopt the vaccines up to 2012 (e.g. Cuba, Costa Rica and Dominican Republic) reported that a different strain (G9P[8]) was the most frequent circulating genotype (>75 %) among their children with diarrhoea [67, 68].

A recent meta-analysis reported that vaccine protection against the G2P[4] strain is lower (39 % in Latin America and 58 % in Europe) than for homotypic and partly-heterotypic strains (>80 % protection) [16], suggesting that the RV1 vaccine may have favoured the selection of this strain in a highly vaccinated population [69]. However, a recent study in Brazil also reported a decrease of G2P[4] incidence from 2011 onwards and that other genotypes, such as G8P[4], G8P[6] and G3P[8] had become more frequent, suggesting that whatever the mechanism underlying these changes, genotype variation is likely to continue after vaccine introduction [70]. Further studies are needed to ascertain if the genotypes in the future represent al strains, of if genotypes for which the vaccines have lower efficacy are over-represented.

Our results should be treated with caution as the reports included have study design limitations as they used descriptive and/or ecological designs, which are not suitable to demonstrate causality. The proportion of cases due to rotavirus and genotype distributions were based on studies with different designs and laboratory methods to identify and characterise rotavirus strains. There was a high heterogeneity among the studies used to calculate the meta-proportion of rotavirus incidence. To counter this heterogeneity, we used the random effects model to minimise its impact on summary estimations. In some locations, the rotavirus proportion and genotype distribution was based on a single study reporting six countries. Countries which have not adopted the vaccine on a large scale, such as Chile and Argentine, allow private practitioners to provide rotavirus vaccinations, which provides services for a selected population of high and middle-income children. Finally, some studies reported data for one year, restricting our ability to describe strain changes over time.

## Conclusions

Post-licensure studies have reported that rotavirus vaccines are effective in preventing rotavirus infection in substantial numbers of children in LA. This evidence strengthens the importance of the vaccines as an effective intervention for reducing the burden of diarrhoea and on rotavirus-specific diarrhoea. Continued surveillance after vaccine introduction is needed to monitor the long-term changes in rotavirus incidence and the potential emergence of heterotypic strains.

## Abbreviations

CI, confidence interval; EIA, enzyme immune-assay; ELISA, enzyme linked immune-assays; LA, Latin America; NOS, Newcastle-Ottawa Scale; OR, odds ratio; RT-PCR, reverse-transcription polymerase chain reaction; RV1, rotarix vaccine; RV5, RotaTeq vaccine; VE, vaccine effectiveness; WHO, World Health Organization

## Additional files

Additional file 1: Multilingual abstracts in the five official working languages of the United Nations. (PDF 412 kb) Additional file 2: Supplementary appendix. (DOCX 240 kb)
