# Supplementary material for: Effectiveness of rotavirus vaccines against rotavirus infection and hospitalization in Latin America: systematic review and meta-analysis
Source: Infect Dis Poverty. 2016 Aug 12;5:83. doi: 10.1186/s40249-016-0173-2 (PMC4982225; doi:10.1186/s40249-016-0173-2)
Supplement: Additional file 1: — Multilingual abstracts in the five official working languages of the United Nations. (PDF 412 kb) [file 40249_2016_173_MOESM1_ESM.pdf]

Translation of the abstract into the five official working languages of the United Nations

مراجعة منهجية وتحليل تلوي للقاحات الفيروسية القهقرية لعلاج الإصابة بالفيروس القهقري والاستشفاء في أمريكا اللاتينية.

Filho, Luis E. Cuevas, Ricardo Q. Gurgel-Victor S. Santos, Daniella P. Marques, Paulo R. S. Martins

موجز

خلفية: كان الفيروس القهقري السبب الرئيسي لدخول الأطفال للمستشفيات في الحالات المرتبطة بالإسهال والوفيات قبل بداية إستعمال لقاحات الفيروس القهقري. الأدوات: تهدف هذه الدراسة لتوصيف فعالية لقاحات الفيروس القهقري لمنع الإصابة بالفيروس القهقري، و تحديد أهم سلالات الفيروس القهقري الموجودة قبل وبعد إدخال اللقاح، من خلال مراجعة منهجية واستعملال التحليل التلوي للدراسات التي نشرت بين عامي 1990 و 2014. تم إدراج 203 دراسة لتقدير نسبة العدوى بسبب الفيروس القهقري و لتقييم عشرة حالات لتأثير اللقاحات. استخدمت 41 دراسة من أصل 46 دراسة في فترة ما بعد التلقيح لإستعمالها في التحليل التلوي للنمط الوراثي، و 20 دراسة لحساب فعالية اللقاح (VE) ضد العدوى، وثمانية دراسات لحساب فعالية اللقاح (VE) ضد المرض بالفيروس وسبعة دراسات لحساب فعالية اللقاح (VE) ضد فيروس القهقري المسبب للإسهال الشديد. النتائج: 24.3٪ من الحالات (95%،= مجال الثقة، 22.1 -26.5) و 16.1٪ (95%= مجال الثقة، 19.3-13.3) من حالات الإسهال بسبب فيروس القهقري قبل وبعد استعمال اللقاح، على التوالي. الفيروسات القهقرية الأكثر انتشارا بعد استعمال اللقاح هم: G2، و51.6% من الحالات بمجال ثقة 95٪(38-65) , و G9 ب 14.5 ٪ من الحالات بمجال ثقة 95٪(7-23)، و G1 ب 14.2٪ من الحالات بمجال ثقة 95٪(7-23)؛ في حين نوع الفيروسات من نوع Pالأكثر انتشارا هي: P 4 من الحالات بمجال ثقة 95٪(41-67) و P8 من الحالات بمجال ثقة 95٪(22-46). وG2P[4] هذا الخليط من النمط الوراثي الأكثر شيوعا بعد استعمال اللقاح. نسبة فعالية اللقاح ضد العدوى هي 53٪من الحالات بمجال ثقة 95٪(64-60) ، 73٪ من الحالات بمجال ثقة 95٪(66-78) ضد المرض بسبب الفيروس و 74٪ من الحالات بمجال ثقة 95٪ (68.0-78.0) ضد الإسهال الشديد. وقد لوحظت تخفيضات في عدد المرضى والوفيات الناجمة عن الإسهال في البلدان التي اعتمدت على استعمال اللقاح الفيروسي المستعمل على الصعيد العالمي. استنتاج: اللقاحات الفيروسية القهقرية فعالة في منع الفيروس القهقري المسبب للإسهال عند الأطفال في أمريكا اللاتينية. هذه اللقاحات مرتبطة بالتغيرات في التوزيع الوراثي.

Translated from English version into Arabic by Aziz Hamdoun, through

TRANSLATORS WITHOUT BORDERS

拉丁美欧轮状病毒疫苗对轮状病毒感染和住院治疗的有效性----系统评价与 Meta 分析

Victor S. Santos, Daniella P. Marques, Paulo R. S. Martins-Filho, Luis E. Cuevas, Ricardo Q. Gurgel

摘要

引言：在轮状病毒疫苗出现以前，轮转病毒是导致儿童腹泻相关入院及死亡的主要原因。

方法：通过系统回顾和荟萃分析 1990 年-2014 年间发表的研究，描述轮状病毒疫苗预防轮状病毒感染和住院的有效性，以及疫苗使用前后流行的主要轮状病毒毒株。被纳入 203 项研究用来估算由轮状病毒引起的感染比例，10 项研究用于评估疫苗的影响。46 项中的 41 项接种后时期研究被用于分析基因型，20 项研究计算了疫苗对感染的有效性，其中 8 个针对住院治疗，7 个针对严重轮状病毒腹泻。

结果：疫苗使用前后，腹泻病例中轮状病毒感染所占比例分别为 24.3% (95%*CI* 22.1-26.5) 和 16.1% (95%*CI* 13.2-19.3)。疫苗使用后，流行最强的轮状病毒 G 型包括 G2 型 (51.6%, 95%*CI* 38-65), G9 型 (14.5%, 95%*CI* 7-23)和 G1 型 (14.2%, 95%*CI* 7-23)，而流行最强的 P 型病毒型为 P[4]型 (54.1%, 95%*CI* 41-67)和 P[8] 型 (33%, 95%*CI* 22-46)，G2P[4]型是最常见的重组型。疫苗对感染、住院治疗和严重腹泻的有效性分别为 53%

(95%*CI* 46-60)、73% (95% *CI*, 66-78)和 74% (95% *CI*, 68.0-78.0)。在普遍使用轮状病毒疫苗的国家中，腹泻相关的入院和死亡率均有所降低。

结论：在拉丁美洲，轮状病毒疫苗可有效预防儿童轮状病毒腹泻。疫苗与基因型分布的变化有关。

Translated from English version into Chinese by Feng Xinyu, edited by Yang Pin, through

Efficacité du vaccin antirotavirus sur les infections à rotavirus et les hospitalisations en Amérique Latine : revue systématique et méta-analyse

Victor S. Santos, Daniella P. Marques, Paulo R. S. Martins-Filho, Luis E. Cuevas, Ricardo Q. Gurgel

Résumé

Contexte : Avant l'introduction des vaccins antirotavirus, les rotavirus étaient la première cause d'hospitalisations et de décès liés à des diarrhées chez l'enfant. **Méthodes :** Au moyen d'une revue systématique et d'une méta-analyse des études publiées entre 1990 et 2014, nous décrivons l'efficacité de la vaccination pour la prévention des infections et hospitalisations causées par le rotavirus et inventorions les principales souches de rotavirus en circulation avant et après l'introduction du vaccin. Nous avons inclus 203 études pour estimer la proportion d'infections dues aux rotavirus et 10 pour évaluer l'impact de la vaccination. Nous avons utilisé 41 études sur les 46 datant de la période antérieure à la vaccination pour une méta-analyse des génotypes, 20 pour calculer l'efficacité de la vaccination contre les infections, 8 pour l'efficacité contre les hospitalisations et 7 pour l'efficacité contre les diarrhées sévères à rotavirus. **Résultats :** 24,3 % (IC à 95 % 22,1-26,5) des cas de diarrhée étaient causés par des rotavirus avant l'introduction de la vaccination et 16,1 % (IC à 95 % 13,2-19,3) après celle-ci. Les types G prévalents après l'introduction des vaccins étaient G2 (51,6 %, IC à 95 % 38-65), G9 (14,5 %, IC à 95 % 7-23) et G1 (14,2 %, IC à 95 % 7-23), tandis que les types P prévalents étaient P[4] (54,1 %, IC à 95 % 41-67) et P[8] (33 %, IC à 95 % 22-46). La combinaison génotypique la plus fréquente après l'introduction du vaccin était G2P[4]. L'efficacité est de 53 % (IC à 95 % 46-60) contre l'infection, 73 % (IC à 95 % 66-78) contre les hospitalisations et 74 % (IC à 95 % 68-78) contre les diarrhées sévères. Des réductions des hospitalisations et de la mortalité causées par la diarrhée ont été observées dans les pays ayant adopté la vaccination universelle contre le rotavirus. **Conclusion :** Les vaccins antirotavirus sont efficaces pour prévenir les diarrhées à rotavirus chez les enfants en Amérique Latine. La vaccination est associée à un changement de la distribution des génotypes.

Translated from English version into French by Suzanne Assenat, through

Эффективность вакцины против инфекции ротавируса и госпитализации в Латинской Америке: систематический обзор и метаанализ

Виктор С. Сантос, Даниэлла П. Маркес, Пауло Р. С. Мартинс-Фильо, Луис Э. Куэвас, Рикардо К. Гургэль

Резюме

Предпосылки: Ротавирус – главная причина госпитализации детей с ротавирусной диареей со смертельным исходом до введения соответствующей вакцины. **Методы:** Мы описываем эффективность антиротавирусной вакцины для предупреждения инфекции и госпитализации, а также рассказываем об основных штаммах ротавируса до и после введения вакцины, на основе систематического обзора и метаанализа исследований, опубликованных за период с 1990 по 2014 год. Было проанализировано 203 исследования для оценки количественного соотношения инфекционных заболеваний, вызванных ротавирусом, и 10 – для определения эффективности вакцинации. Из 46 исследований, проведенных в поствакцинальный период, 41 было использовано для метаанализа генотипов, 20 – для рассчета эффективности вакцины против инфекции, 8 – для рассчета эффективности вакцины против госпитализации и 7 – против тяжелых случаев ротавирусной диареи. **Результаты:** Зафиксировано 24,3% (95% ДИ 22,1-26,5) и 16,1% (95% ДИ 13,2-19,3) случаев ротавирусной диареи соответственно до и после проведения вакцинации. Преобладающие генотипы G после введения вакцины: G2 (51,6%, 95% ДИ 38-65), G9 (14,5%, 95% ДИ 7-23) и G1 (14,2%, 95% ДИ 7-23), тогда как среди генотипов P превалирующими стали P[4] (54,1%, 95% ДИ 41-67) и P[8] (33%, 95% ДИ 22-46). Комбинация генотипов G2P[4] оказалась самой распространенной после внедрения вакцины. Действенность вакцины составила 53% (95% ДИ 46-60) против инфекции, 73% (95% ДИ 66-78) – против госпитализации и 74% (95% ДИ 68-78) – против тяжелых случаев диареи. В странах, где проводилась всеобщая антиротавирусная вакцинация, наблюдалось уменьшение числа случаев госпитализации и смертельного исхода, вызванных ротавирусной диареей. **Заключение:** Антиротавирусная вакцина эффективна для предотвращения ротавирусной диареи у детей в Латинской Америке. Вакцина обуславливает изменения в распределении генотипов.

Translated from English version into Russian by Elena Surina, through

TRANSLATORS
WITHOUT BORDERS

Eficacia de las vacunas contra la infección por rotavirus y hospitalización en América Latina: meta-análisis y revisión sistemática

Victor S. Santos, Daniella P. Marques, Paulo R. S. Martins-Filho, Luis E. Cuevas, Ricardo Q. Gurgel

Resumen

Antecedentes: El rotavirus fue la causa principal de hospitalizaciones por diarrea y mortalidad en niños previo a la introducción de las vacunas contra el rotavirus. **Métodos:** Describimos la eficacia de las vacunas contra el rotavirus en la prevención de infecciones por rotavirus y hospitalizaciones y las principales cepas de rotavirus que circulaban antes y después de la introducción de la vacuna en una revisión sistemática y meta-análisis de los estudios publicados entre los años 1990 y 2014. Se incluyeron 203 estudios para el cálculo de la proporción de infecciones a causa de rotavirus y 10 para evaluar el impacto de las vacunas. Se utilizaron 41 de 46 estudios en el período post-vacuna

para realizar un meta-análisis de genotipos, 20 para calcular la eficacia de las vacunas contra la infección, ocho para calcular la eficacia de las vacunas contra la hospitalización y siete para calcular la eficacia de las vacunas contra la diarrea severa por rotavirus. **Resultados:** 24,3% (95% IC 22,1-26,5) y 16,1% (95% IC 13,2-19,3) de los casos de diarrea fueron a causa de rotavirus antes y después de la introducción de la vacuna, respectivamente. Los tipos G más prevalentes luego de la introducción de la vacuna fueron G2 (51,6%, 95% IC 38-65), G9 (14,5%, 95% IC 7-23) y G1 (14,2%, 95% IC 7-23); mientras que los tipos P más prevalentes fueron P[4] (54,1%, 95% IC 41-67) y P[8] (33%, 95% IC 22-46). G2P[4] fue la combinación de genotipo más frecuente con posterioridad a la introducción de la vacuna. La eficacia fue del 53% (95% IC 46-60) contra la infección,73% (95% IC, 66-78) contra la hospitalización y 74% (95% IC, 68,0-78,0) contra la diarrea severa. Se observó una disminución en la cantidad de internaciones y mortalidad a causa de diarrea en los países que adoptaron la vacuna contra el rotavirus de manera universal. **Conclusión:** Las vacunas contra el rotavirus son eficaces en la prevención de la diarrea por rotavirus en niños en América Latina. Las vacunas se asociaban a cambios en la distribución del genotipo.

Translated from English version into Spanish by Maria Alejandra Aguada, through

TRANSLATORS
WITHOUT BORDERS
