# Supplementary material for: Effectiveness of rotavirus vaccines against rotavirus infection and hospitalization in Latin America: systematic review and meta-analysis
Source: Infect Dis Poverty. 2016 Aug 12;5:83. doi: 10.1186/s40249-016-0173-2 (PMC4982225; doi:10.1186/s40249-016-0173-2)
Supplement: Additional file 2: — Supplementary appendix. (DOCX 240 kb) [file 40249_2016_173_MOESM2_ESM.docx]

**Supplementary appendix**

**Search strategy PubMed**

| Search | Query | Itens found |
| --- | --- | --- |
| [#1](http://www.ncbi.nlm.nih.gov/pubmed/advanced) | Search[All Fields] AND "Rotavirus"[MeSH] OR ("rotavirus"[MeSH Terms] OR "rotavirus"[All Fields] OR "rotaviruses"[All Fields]) OR (neonatal[All Fields] AND calf[All Fields] AND ("diarrhoea"[All Fields] OR "diarrhea"[MeSH Terms] OR "diarrhea"[All Fields]) AND ("viruses"[MeSH Terms] OR "viruses"[All Fields] OR "virus"[All Fields])) OR "Rotavirus Infections"[MeSH] AND ("rotavirus infections"[MeSH Terms] OR ("rotavirus"[All Fields] AND "infections"[All Fields]) OR "rotavirus infections"[All Fields] OR ("infection"[All Fields] AND "rotavirus"[All Fields])) OR ("rotavirus infections"[MeSH Terms] OR ("rotavirus"[All Fields] AND "infections"[All Fields]) OR "rotavirus infections"[All Fields] OR ("infections"[All Fields] AND "rotavirus"[All Fields])) OR ("rotavirus infections"[MeSH Terms] OR ("rotavirus"[All Fields] AND "infections"[All Fields]) OR "rotavirus infections"[All Fields] OR ("rotavirus"[All Fields] AND "infection"[All Fields]) OR "rotavirus infection"[All Fields]) AND "Rotavirus Vaccines"[MeSH] OR ("rotavirus vaccines"[MeSH Terms] OR ("rotavirus"[All Fields] AND "vaccines"[All Fields]) OR "rotavirus vaccines"[All Fields] OR ("vaccines"[All Fields] AND "rotavirus"[All Fields])) AND (("1990/01/01"[PDAT] : "2014/09/30"[PDAT]) AND "humans"[MeSH Terms]) | **2,191** |

**Search strategy SCOPUS**

| Search | Query | Itens found |
| --- | --- | --- |
| [#1](http://www.ncbi.nlm.nih.gov/pubmed/advanced) | ( title-abs-key ( rotavirus ) or title-abs-key ( rotavirus infections ) and title-abs-key ( rotavirus vaccines ) ) and pubyear > 1989 and pubyear < 2015 | **4,895** |

**Search strategy LILACS**

| Search | Query | Itens found |
| --- | --- | --- |
| [#1](http://www.ncbi.nlm.nih.gov/pubmed/advanced) | (mh:"Rotavirus") OR (mh: B04.820.630.790$) OR (mh: B04.909.777.714.790$) OR (mh: SP4.011.107.268.444.755$) OR (mh:“Infecções por Rotavirus”) OR (mh:” Rotavirus Infections”) OR (mh:“Infecciones por Rotavirus”) OR (mh:C02.782.791.814$)) AND ((mh:“Vacinas contra Rotavirus”) OR (vacinas) OR (vacinas candidatas usadas para prevenir infecção com Rotavirus) OR (mh:“Rotavirus Vaccines”) OR (mh:“Vacinas contra Rotavirus”) OR (mh:D20.215.894.899.760$)) | **65** |

**Table S1. Study characteristics.**

| **Study** | **Year of publication** | **Period** | | **Country** | **Type of study** | **Setting** | **Sample size** | **Rotavirus-positive** |
| --- | --- | --- | --- | --- | --- | --- | --- | --- |
|  |  | **Start** | **End** |  |  |  |  |  |
| Linhares 1990 | 1990 | 1988 | 1988 | Brazil | - | H | 62 | 4 |
| De la Cruz 1990 | 1990 | 1983 | 1984 | Cuba | CS | H | 256 | 72 |
| Cruz 1990 | 1990 | 1987 | 1988 | Guatemala | CH | H | 442 | 49 |
| Cravioto 1990 | 1990 | 1995 | 1987 | Mexico | CC | H | 314 | 13 |
| Penny 1990 | 1990 | 1985 | 1987 | Peru | CS | H | 38 | 10 |
| Perez-Schael 1990 | 1990 | 1985 | 1986 | Venezuela | - | H | 223 | 22 |
| Caillou 1991 | 1991 | 1990 | 1991 | Argentina | NA | H | 362 | 22 |
| Rosin de Castagnaro 1991 | 1991 | 1990 | 1990 | Argentina | CS | H | 282 | 152 |
| Gomes 1991 | 1991 | 1985 | 1986 | Brazil | CC | H | 500 | 70 |
| Stewien 1991 | 1991 | 1986 | 1988 | Brazil | CS | H | 479 | 91 |
| Teixeira 1991 | 1991 | 1986 | 1990 | Brazil | CS | H | 495 | 129 |
| Huilan 1991 | 1991 | 1982 | 1985 | Mexico | CC | H | 559 | 73 |
| Brown 1991 | 1991 | 1986 | 1989 | Peru | CS | H | 116 | 26 |
| Greenberg 1991 | 1991 | 1985 | 1987 | Peru | CS | H | 76 | 21 |
| Pazzaglia 1991 | 1991 | 1988 | 1989 | Peru | CS | H | 391 | 70 |
| Stephensen 1991 | 1991 | 1990 | 1991 | Peru | CS | H | 708 | 163 |
| White 1991 | 1991 | 1979 | 1989 | Venezuela | CS | H | 2056 | 617 |
| Vergara 1992 | 1992 | 1986 | 1989 | Argentina | CS | H | 972 | 68 |
| Brunser 1992 | 1992 | 2000 | 2004 | Chile | - | H | 115 | 26 |
| Lanata 1992 | 1992 | 1985 | 1987 | Peru | CC | H | 2780 | 111 |
| Maldonado 1992 | 1992 | 1987 | 1987 | Venezuela | CS | H | 511 | 87 |
| Linhares 1993 | 1993 | 1988 | 1988 | Brazil | CS | H | 101 | 39 |
| Pereira 1993 | 1993 | 1981 | 1990 | Brazil | Surveillance | H | 11172 | 1788 |
| Regua 1993 | 1993 | 1987 | 1988 | Brazil | CS | H | 406 | 49 |
| Stewien 1993 | 1993 | 1988 | 1989 | Brazil | CS | H | 67 | 13 |
| Timenetsky 1993 | 1993 | 1987 | 1988 | Brazil | CS | H | 97 | 11 |
| Estevez 1993 | 1993 | 1991 | 1991 | Cuba | CS | H | 4383 | 351 |
| Flores-Abuxapqui 1993 | 1993 | 1991 | 1991 | Mexico | CS | H | 105 | 15 |
| Velazquez 1993 | 1993 | 1987 | 1989 | Mexico | CH | H | 962 | 87 |
| Miranda 1993 | 1993 | 1991 | 1987 | Peru | CS | H | 82 | 34 |
| Salazar-Lindo 1993 | 1993 | 1987 | 1987 | Peru | CS | H | 42 | 18 |
| Figueroa 1993 | 1993 | 1990 | 1991 | Peru | CS | H | 249 | 82 |
| Ludert 1993 | 1993 | 1991 | 1992 | Venezuela | CS | H | 214 | 49 |
| Rosin de Castagnaro 1994 | 1994 | 1985 | 1989 | Argentina | CS | H | 1308 | 353 |
| Gonzalez-Losa 1994 | 1994 | 1985 | 1990 | Mexico | CS | H | 827 | 174 |
| Callejas 1994 | 1994 | 1989 | 1991 | Venezuela | CS | H | 200 | 28 |
| Borsa 1995 | 1995 |  |  | Argentina | NA | H | 1236 | 445 |
| O'Ryan 1995 | 1995 | 2004 | 2005 | Chile | CS | H | 1026 | 523 |
| Contreras 1995 | 1995 | 1993 | 1994 | Mexico | CS | H | 465 | 140 |
| Vergara 1996 | 1996 |  |  | Argentina | CC | H | 95 | 9 |
| Chea-Woo 1996 | 1996 | 1991 | 1996 | Peru | CS | H | 307 | 117 |
| Lanata 1996 | 1996 | 1987 | 1990 | Peru | - | H | 2115 | 85 |
| O'Ryan 1997 | 1997 | 1985 | 1987 | Chile | CS | H | 1841 | 736 |
| Espinoza 1997 | 1997 | 1994 | 1994 | Nicaragua | Case series | H | 435 | 52 |
| Espinoza 1997 | 1997 | 1994 | 1994 | Nicaragua | CS | H | 296 | 83 |
| Perez-Schael 1997 | 1997 | 1988 | 1993 | Venezuela | - | H | 808 | 137 |
| Teixeira 1998 | 1998 | 1994 | 1994 | Brazil | CS | H | 407 | 106 |
| Guerrero 1998 | 1998 | 1988 | 1991 | Mexico | CH | H | 510 | 26 |
| Maldonado 1998 | 1998 | 1992 | 1995 | Mexico | CS | H | 305 | 12 |
| Padilla Noriega 1998 | 1998 | 1995 | 1996 | Mexico | CS | H | 1696 | 577 |
| Maldonado 1998 | 1998 | 1992 | 1993 | Venezuela | CS | H | 321 | 161 |
| Uriarte 1999 | 1999 | 1994 | 1995 | Argentina | CS | H | 134 | 52 |
| Gusmao 1999 | 1999 | 1992 | 1994 | Brazil | CH | H | 181 | 38 |
| Correa 1999 | 1999 | 1995 | 1996 | Colombia | CS | H | 131 | 42 |
| Mattar 1999 | 1999 | 1997 | 1997 | Colombia | CS | H | 471 | 94 |
| Cama 1999 | 1999 | 1995 | 1997 | Peru | Case series | H | 762 | 335 |
| Callejas 1999 | 1999 | 1993 | 1995 | Venezuela | CC | H | 378 | 60 |
| Vizcaya Delgado 1999 | 1999 | 1993 | 1995 | Venezuela | CC | H | 613 | 74 |
| Arguelles 2000 | 2000 | 1996 | 1998 | Argentina | CS | H | 500 | 310 |
| Espul 2000 | 2000 | 1995 | 1996 | Argentina | CS | H | 957 | 153 |
| Bittencourt 2000 | 2000 | 1996 | 1998 | Brazil | CS | H | 603 | 96 |
| Da Silva Domingues 2000 | 2000 | 1995 | 1996 | Brazil | CS | H | 102 | 16 |
| Fernandes 2000 | 2000 | 1996 | 1998 | Brazil | CS | H | 1903 | 152 |
| Gonzalez-Losa 2000 | 2000 | 1989 | 1990 | Mexico | CS | H | 149 | 25 |
| Bok 2001 | 2001 | 1996 | 1998 | Argentina | CS | H | 1312 | 551 |
| Giordano 2001 | 2001 | 1997 | 1998 | Argentina | Case series | H | 133 | 47 |
| O'Ryan 2001 | 2001 | 1997 | 1999 | Argentina | Surveillance | H | 1167 | 467 |
| Cardoso 2001 | 2001 | 1986 | 1995 | Brazil | CS | H | 1979 | 237 |
| Da Rosa e Silva 2001 | 2001 | 1998 | 1998 | Brazil | CS | H | 656 | 79 |
| Orlandi 2001 | 2001 | 1998 | 1999 | Brazil | CC | H | 130 | 25 |
| O'Ryan 2001 | 2001 | 1993 | 1995 | Chile | Surveillance | H | 2015 | 705 |
| Mota-Hernandez 2001 | 2001 | 1994 | 1995 | Mexico | CS | H | 520 | 265 |
| Ramirez Del Puerto 2001 | 2001 | 1997 | 1998 | Uruguay | CS | H | 120 | 55 |
| O'Ryan 2001 | 2001 | 1997 | 1999 | Venezuela | Surveillance | H | 3875 | 1201 |
| Araujo 2002 | 2002 | 1996 | 1998 | Brazil | CS | H | 619 | 93 |
| Rosa e Silva 2002 | 2002 | 1998 | 1999 | Brazil | CS | H | 1056 | 95 |
| Soares 2002 | 2002 | 1998 | 2000 | Brazil | CS | H | 1613 | 194 |
| Rodriguez Angulo 2002 | 2002 | 2000 | 2000 | Mexico | CS | H | 668 | 334 |
| Coluchi 2002 | 2002 | 1999 | 2000 | Paraguay | CS | H | 220 | 70 |
| Cardoso 2003 | 2003 | 1986 | 2000 | Brazil | CS | H | 1324 | 357 |
| Da Silva 2003 | 2003 | 1999 | 2000 | Brazil | CS | H | 485 | 34 |
| Santos 2003 | 2003 | 1997 | 1999 | Brazil | CS | H | 678 | 156 |
| Urbina 2003 | 2003 | 1998 | 2000 | Colombia | CS | H | 228 | 89 |
| Carmona 2004 | 2004 | 1994 | 1995 | Brazil | CS | H | 117 | 33 |
| Costa 2004 | 2004 | 2000 | 2002 | Brazil | Case series | H | 207 | 77 |
| Agudelo 2004 | 2004 | 2002 | 2002 | Colombia | CS | H | 1335 | 93 |
| Guardado 2004 | 2004 | 2001 | 2002 | El Salvador | Case series | H | 322 | 87 |
| Guerrero 2004 | 2004 | 1998 | 1999 | Mexico | CH | H | 169 | 15 |
| Salinas 2004 | 2004 | 1998 | 2002 | Venezuela | Surveillance | H | 11988 | 2757 |
| Cuestas Montañes 2005 | 2005 | 2002 | 2003 | Argentina | CS | H | 73 | 33 |
| Carneiro 2005 | 2005 | 2003 | 2004 | Brazil | Case series | H | 2105 | 211 |
| Da Luz 2005 | 2005 | 1997 | 1999 | Brazil | Case series | H | 128 | 41 |
| Santos 2005 | 2005 | 1999 | 2002 | Brazil | Surveillance | H | 648 | 207 |
| Valois 2005 | 2005 | 1986 | 1992 | Brazil | RCT | H | 90 | 55 |
| Caceres 2005 | 2005 | 2000 | 2001 | Colombia | CC | H | 290 | 125 |
| Noyola 2005 | 2005 | 1998 | 2004 | Mexico | CS | H | 3716 | 1784 |
| Martínez 2005 | 2005 | 2001 | 2004 | Paraguay | CS | H | 533 | 91 |
| Parra 2005 | 2005 | 1998 | 2000 | Paraguay | CS | H | 410 | 94 |
| Castello 2006 | 2006 | 1999 | 2003 | Argentina | Surveillance | H | 1212 | 182 |
| Protegiendo la Salud de las Américas 2006 | 2006 | 2005 | 2006 | Bolivia | Surveillance | H | 322 | 45 |
| Barreto 2006 | 2006 | 2001 | 2005 | Brazil | Surveillance | H | 139 | 11 |
| Carvalho-Costa 2006 | 2006 | 2004 | 2004 | Brazil | CS | H | 134 | 64 |
| Compagnoli Carmona 2006 | 2006 | 1996 | 2003 | Brazil | CS | H | 3101 | 775 |
| Pietruchinski 2006 | 2006 | 2003 | 2003 | Brazil | CS | H | 251 | 70 |
| Volotao 2006 | 2006 |  |  | Brazil | Surveillance | H | 1568 | 125 |
| Caceres 2006 | 2006 | 2003 | 2004 | Colombia | CS | H | 726 | 356 |
| Gutierrez 2006 | 2006 | 1999 | 2000 | Colombia | CS | H | 300 | 39 |
| Manrique 2006 | 2006 | 2004 | 2004 | Colombia | CS | H | 129 | 62 |
| Protegiendo la Salud de las Américas 2006 | 2006 | 2005 | 2005 | Honduras | Surveillance | H | 597 | 72 |
| Romero 2007 | 2007 | 2001 | 2002 | Bolivia | CS | H | 1163 | 221 |
| Andreasi 2007 | 2007 | 2000 | 2004 | Brazil | CS | H | 380 | 87 |
| Gouvea 2007 | 2007 | 2002 | 2006 | Brazil | CS | H | 192 | 96 |
| Magalhaes 2007 | 2007 | 2000 | 2002 | Brazil | CC | H | 470 | 113 |
| Montenegro 2007 | 2007 | 2004 | 2005 | Brazil | CS | H | 290 | 102 |
| Serravalle 2007 | 2007 | 2000 | 2004 | Brazil | CS | H | 358 | 168 |
| Soares 2007 | 2007 | 1998 | 2005 | Brazil | CS | H | 2421 | 242 |
| O'Ryan 2007 | 2007 |  |  | Chile | Surveillance | H | 1313 | 184 |
| Inciensa 2007 | 2007 | 2005 | 2005 | Costa Rica | Surveillance | H | 151 | 53 |
| Endara 2007 | 2007 | 2003 | 2006 | Ecuador | CS | H | 411 | 95 |
| Ruiz Palacios 2007 | 2007 | 2001 | 2003 | Mexico | RCT | H | 97 | 18 |
| Amarilla 2007 | 2007 | 2004 | 2005 | Paraguay | CS | H | 1713 | 377 |
| Parra 2007 | 2007 | 2002 | 2005 | Paraguay | CS | H | 1588 | 445 |
| Giordano 2008 | 2008 | 1977 | 2002 | Argentina | CS | H | 2224 | 423 |
| PAHO 2008 | 2008 | 2005 | 2007 | Bolivia | CS | H | 2982 | 1133 |
| Carraro 2008 | 2008 | 2003 | 2005 | Brazil | CS | H | 3768 | 754 |
| Domingues 2008 | 2008 | 2005 | 2006 | Brazil | Case series | H | 260 | 39 |
| Martini 2008 | 2008 | 2003 | 2004 | Brazil | CS | H | 328 | 95 |
| Ribeiro 2008 | 2008 | 2004 | 2006 | Brazil | CS | H | 68 | 14 |
| Delpiano 2008 | 2008 | 1983 | 1984 | Chile | Surveillance | H | 2413 | 338 |
| PAHO 2008 | 2008 | 2006 | 2008 | Chile | CS | H | 1230 | 271 |
| Bourdett-Stanziola 2008 | 2008 | 2002 | 2003 | Costa Rica | CS | H | 680 | 360 |
| Bourdett-Stanziola 2008 | 2008 | 2002 | 2003 | Dominican Republic | CS | H | 402 | 249 |
| Naranjo 2008 | 2008 | 2006 | 2006 | Ecuador | CS | H | 729 | 270 |
| PAHO 2008 | 2008 | 2005 | 2007 | El Salvador | CS | H | 6085 | 2312 |
| PAHO 2008 | 2008 | 2005 | 2007 | Guatemala | CS | H | 4212 | 2148 |
| PAHO 2008 | 2008 | 2005 | 2007 | Guyana | CS | H | 384 | 31 |
| PAHO 2008 | 2008 | 2005 | 2007 | Honduras | CS | H | 5956 | 2204 |
| PAHO 2008 | 2008 | 2009 | 2009 | Nicaragua | CS | H | 1186 | 261 |
| Bourdett-Stanziola 2008 | 2008 | 2002 | 2003 | Panamá | CS | H | 1007 | 473 |
| PAHO 2008 | 2008 | 2009 | 2009 | Paraguay | CS | H | 920 | 386 |
| PAHO 2008 | 2008 | 2009 | 2009 | St.Vincent | CS | H | 131 | 33 |
| PAHO 2008 | 2008 | 2009 | 2009 | Surinam | CS | H | 256 | 87 |
| González 2008 | 2008 | 2006 | 2007 | Venezuela | CS | H | 160 | 30 |
| Villalobos 2008 | 2008 | 2004 | 2004 | Venezuela | CS | H | 90 | 21 |
| Stupka 2009 | 2009 | 2006 | 2007 | Argentina | CS | H | 2714 | 733 |
| PAHO 2009 | 2009 | 2008 | 2008 | Bolivia | CS | H | 1501 | 720 |
| Carvalho-Costa 2009 | 2009 | 2005 | 2007 | Brazil | CS | H | 464 | 135 |
| O'Ryan 2009 | 2009 | 1997 | 1999 | Chile | CH | H | 145 | 20 |
| PAHO 2009 | 2009 | 2003 | 2003 | Chile | CS | H | 1082 | 292 |
| PAHO 2009 | 2009 | 2008 | 2008 | Guatemala | CS | H | 1190 | 393 |
| PAHO 2009 | 2009 | 2008 | 2008 | Honduras | CS | H | 1907 | 687 |
| Patel 2009 | 2009 | 2007 | 2008 | Nicaragua | CC | H+C | 1589 | 286 |
| PAHO 2009 | 2009 | 2008 | 2008 | Nicaragua | CS | H | 1638 | 278 |
| PAHO 2009 | 2009 | 2008 | 2008 | Panamá | CS | H | 567 | 45 |
| PAHO 2009 | 2009 | 2008 | 2008 | Paraguay | CS | H | 263 | 42 |
| Perez-Schael 2009 | 2009 | 1993 | 1995 | Venezuela | Case series | H | 6742 | 1820 |
| Ministerio de Salud Pública |  | 2004 | 2004 | Uruguay | CS | H | 214 | 64 |
| Gurgel, 2007 | 2007 | 2006 | 2007 | Brazil | CS | H | 129 | 21 |
| Nakagomi, 2008 | 2008 | 2006 | 2007 | Brazil | CS | H | 470 | 70 |
| Carvalho-Costa, 2009 | 2009 | 2006 | 2007 | Brazil | CS | H | 197 | 46 |
| Gurgel, 2009 | 2009 | 2006 | 2008 | Brazil | CS | H | 534 | 59 |
| Munford, 2009 | 2009 | 2006 | 2006 | Brazil | CS | H | 402 | 191 |
| Correia, 2010 | 2010 | 2006 | 2008 | Brazil | CS | H | 926 | 119 |
| Esteban, 2010 | 2010 | 2006 | 2007 | Argentina | CS | H | 292 | 49 |
| Maldonado, 2010 | 2010 | 2006 | 2007 | Venezuela | CS | H | 241 | 47 |
| Marillo, 2010 | 2010 | 2006 | 2008 | Brazil | CS | - | 62 | 13 |
| Mascarenhas, 2010 | 2010 | 2006 | 2008 | Brazil | CS | H | 241 | 16 |
| Nunes, 2010 | 2010 | 2006 | 2007 | Brazil | CS | C | 124 | 31 |
| Sáfadi, 2010 | 2010 | 2006 | 2008 | Brazil | CS | H | 204 | 36 |
| Silva, 2010 | 2010 | 2007 | 2008 | Brazil | CS | H | 171 | 33 |
| Borges, 2011 | 2011 | 2008 | 2008 | Brazil | CS | C | 220 | 8 |
| Carvalho-Costa, 2011 | 2011 | 2006 | 2009 | Brazil | CS | H | 4817 | 908 |
| Cilli, 2011 | 2011 | 2006 | 2009 | Brazil | CS | C | 320 | 80 |
| Gómez, 2011 | 2011 | 2006 | 2009 | Brazil | CS | H | 75 | 75 |
| Justino, 2011 | 2011 | 2008 | 2009 | Brazil | CC | H | 1391 | 538 |
| Vieira, 2011 | 2011 | 2006 | 2008 | Brazil | CH | C | 444 | 16 |
| Yen, 2011 | 2011 | 2010 | 2010 | Mexico | CC | H | 56 | 16 |
| Dulgheroff, 2012 | 2012 | 2007 | 2010 | Brazil | CS | H+C | 630 | 76 |
| O'Ryan, 2012 | 2012 | 2009 | 2010 | Chile | CH | H | 967 | 296 |
| Assis, 2013 | 2013 | 2006 | 2011 | Brazil | CS | H | 529 | 54 |
| Gómez, 2013 | 2013 | 2009 | 2010 | Brazil | CS | H | 6 | 6 |
| Lopman, 2013 | 2013 | 2011 | 2012 | Ecuador | CC | C | 404 | 76 |
| Pereira, 2013 | 2013 |  |  | Brazil | - | - | 198 | 30 |
| Roig, 2013 | 2013 | 2009 | 2010 | Argentina | CS | H | 275 | 51 |
| Cotes-Cantillo, 2014 | 2014 | 2011 | 2013 | Colombia | CC | H+C | 1051 | 193 |
| Espejo, 2014 | 2014 | 2012 | 2012 | Peru | CH | H | 117 | 42 |
| Ichiara, 2014 | 2014 | 2008 | 2011 | Brazil | CC | H | 2176 | 215 |
| Mandile, 2014 | 2014 | 2008 | 2011 | Argentina | CS | H | 663 | 139 |
| Peláez-Carvajal, 2014 | 2014 | 2008 | 2012 | Colombia | CS | H | 467 | 467 |
| Sandra, 2014 | 2014 | 2006 | 2008 | Brazil | CS | - | 591 | 103 |
| Soares, 2014 | 2014 | 2011 | 2012 | Brazil | CH | H | 764 | 263 |
| de Palma, 2010 | 2010 | 2007 | 2009 | El Salvador | CC | H+C | 2061 | 323 |
| Patel, 2011 | 2011 | 2006 | 2009 | El Salvador | CS | H | 8287 | 1635 |
| Patel, 2013 | 2013 | 2010 | 2011 | Bolivia | CS | H+C | 2318 | 400 |
| Patel, 2009 | 2009 | 2007 | 2008 | Nicaragua | CC | H+C | 1615 | 285 |
| Becker-Dreps, 2011 | 2011 | 2008 | 2009 | Nicaragua | CS | C | 392 | 14 |
| Mast, 2011 | 2011 | 2007 | 2009 | Nicaragua | CC | H+C | 6174 | 1082 |
| Bucardo, 2012 | 2012 | 2010 | 2010 | Nicaragua | CS | H | 107 | 18 |
| García-Puebla, 2012 | 2012 | 2007 | 2008 | Puerto Rico | CS | H | 7686 | 1199 |
| Patel, 2012 | 2012 | 2007 | 2010 | Nicaragua | CC | H | 11573 | 1016 |
| Becker-Dreps, 2014 | 2014 | 2010 | 2011 | Nicaragua | CH | C | 826 | 18 |
| Bucardo, 2014 | 2014 | 2009 | 2010 | Nicaragua | CS | H+C | 330 | 25 |
| Khawaja, 2014 | 2014 | 2007 | 2009 | Nicaragua | CS | H | 6064 | 1082 |
| **Studies reporting vaccine impact** |  |  |  |  |  |  |  |  |
| do Carmo, 2011 | 2011 | 2002 | 2009 | Brazil | Ec | H | N/A | N/A |
| Gurgel, 2011 | 2011 | 2002 | 2009 | Brazil | Ec | H | N/A | N/A |
| Lanzieri, 2011 | 2011 | 2004 | 2008 | Brazil | Ec | H | N/A | N/A |
| Richardson, 2010 | 2010 | 2003 | 2008 | Mexico | Ec | H | N/A | N/A |
| Gastañaduy, 2013 | 2013 | 2003 | 2011 | Mexico | Ec | H | N/A | N/A |
| Bayard, 2012 | 2012 | 2000 | 2008 | Panama | Ec | H | N/A | N/A |
| Masukawa, 2014 | 2014 | 2000 | 2011 | Brazil | Ec | H | N/A | N/A |
| Fernandes, 2014 | 2014 | 2000 | 2011 | Brazil | Ec | H | N/A | N/A |
| Yen, 2011 | 2011 | 2005 | 2009 | El Salvador | Ec | H | N/A | N/A |
| Quintanar-Solares, 2011 | 2011 | 2003 | 2009 | Mexico | Ec | H | N/A | N/A |
| Esparza-Aguilar, 2014 | 2014 | 2003 | 2011 | Mexico | Ec | H | N/A | N/A |
| Molto, 2011 | 2011 | 2000 | 2008 | Panama | Ec | H | N/A | N/A |

RV1= Rotarix vaccine; RV5= Rotateq vaccine; CS= cross-sectional study; CC= case-control study; CH= cohort study; Ec= ecological study; NOS= New Castle-Ottawa scale; H= hospital; H+C= hospital + community; C= community.

**Table S2. Results of meta-regression by using mixed-effects model to explore sources of heterogeneity across studies.**

| **Variables** | **p-value (test for subgroup differences)** | **Tau^2^** | **Adjusted R^2^** |
| --- | --- | --- | --- |
| Vaccine type | 0.790 | 0.047 | 0 |
| Country income | 0.614 | 0.031 | 0 |
| Setting | 0.438 | 0.035 | 0 |
| Latitude | 0.258 | 0.022 | 22.3 |
| Vaccination coverage | 0.906 | 0.033 | 0 |

Tau^2^, estimated amount of residual heterogeneity; R^2^, % residual variation due to heterogeneity.

**Figures**


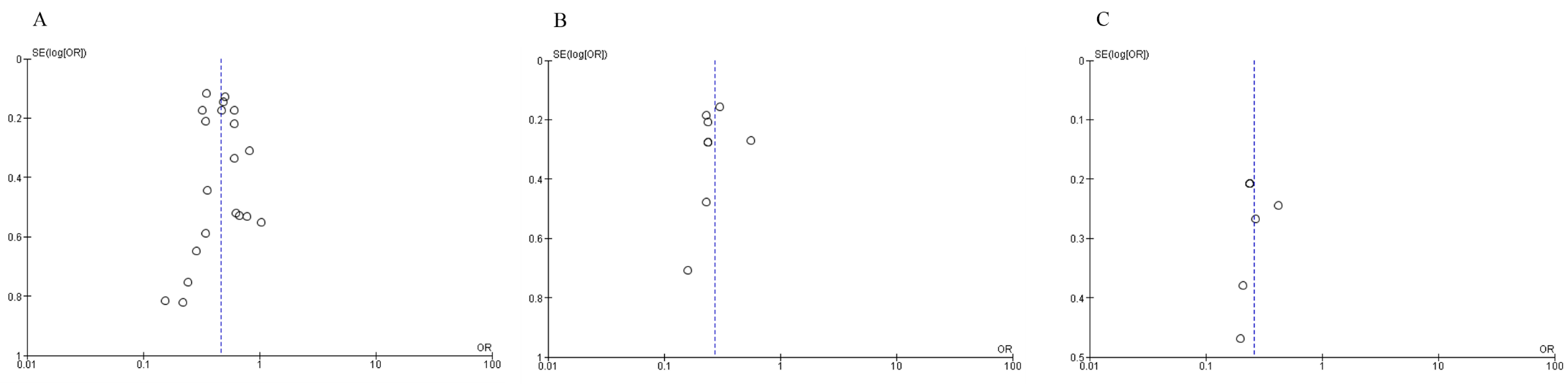


**Figure S1**. Funnel plot for the overall analysis of vaccine protection (A) and effectiveness of rotavirus vaccine against rotavirus hospitalizations (B) and severe diarrhoea (C).
